# Renal Tube Casts without Albuminuria

**Published:** 1908-11-28

**Authors:** 


### RENAL TUBE CASTS WITHOUT ALBUMINURIA.

It has to be borne in mind how easy the detection of casts has been made by the use of the centrifugal machine, so that too much stress must not be attached to the presence of a few casts in the carefully centrifugalised deposit from a urine. Like albumin, they may occur under a number of circumstances in the urine of persons whose condition is little or not at all abnormal.

Renal tube casts are thus at times met with in normal infants during the first week of life, and in normal women at some time during pregnancy or parturition. Considerable judgment is needed in attempting to interpret the significance of the presence or absence of casts in the urine. They may be present when there is no serious kidney lesion, and they may be few in number or absent when the kidneys are markedly diseased.

It is generally supposed that the absence of albumin from a specimen of urine necessarily implies that no casts are present either. This is very far from being the case. Urine containing casts but no serum-albumin has been observed in many diseases. This condition may occur when there is but slight and transient irritation of the renal epithelium. In may even occur in conditions of complete health, as in infants during the first few days of life. It has been carefully studied by Dr. C. M. Montgomery in several hundreds of consecutive cases of pulmonary tuberculosis, recorded in the fourth annual report of the Henry Phipps Institute. In 398 cases examined during one year, 129 showed casts. The latter were in association with albumin seventy-two times (18 per cent.), and they were present without any albumin at all fifty-seven times (14 per cent.).

Even when albumin and casts are present in the same specimen the amount of the former and the number of the latter in many cases do not run parallel with each other. A patient with one part per 1,000 of albumin may show only a few casts, and another patient with no albumin may exhibit many casts, even of the granular type. In regard to the possible absence of albumin when many casts are present Montgomery's results agree with those of Kossler (*Berl. Klin. Wochenschr.*, Vol. xxxii., 1895, p. 296), who reported eighteen cases with casts but without serum-albumin in the urine.

